# Aryl Hydrocarbon Receptor in Atopic Dermatitis and Psoriasis

**DOI:** 10.3390/ijms20215424

**Published:** 2019-10-31

**Authors:** Masutaka Furue, Akiko Hashimoto-Hachiya, Gaku Tsuji

**Affiliations:** 1 Department of Dermatology, Graduate School of Medical Sciences, Kyushu University, Maidashi 3-1-1, Higashiku, Fukuoka 812-8582, Japan; ahachi@dermatol.med.kyushu-u.ac.jp (A.H.-H.); gakku@dermatol.med.kyushu-u.ac.jp (G.T.); 2 Research and Clinical Center for Yusho and Dioxin, Kyushu University, Maidashi 3-1-1, Higashiku, Fukuoka 812-8582, Japan; 3 Division of Skin Surface Sensing, Graduate School of Medical Sciences, Kyushu University, Maidashi 3-1-1, Higashiku, Fukuoka 812-8582, Japan

**Keywords:** aryl hydrocarbon receptor (AHR), aryl hydrocarbon receptor-nuclear translocator (ARNT), nuclear factor-erythroid 2-related factor-2 (NRF2), atopic dermatitis, psoriasis, tapinarof, filaggrin, skin barrier, Th17, Th22, Treg, reactive oxygen species, antioxidants

## Abstract

The aryl hydrocarbon receptor (AHR)/AHR-nuclear translocator (ARNT) system is a sensitive sensor for small molecular, xenobiotic chemicals of exogenous and endogenous origin, including dioxins, phytochemicals, microbial bioproducts, and tryptophan photoproducts. AHR/ARNT are abundantly expressed in the skin. Once activated, the AHR/ARNT axis strengthens skin barrier functions and accelerates epidermal terminal differentiation by upregulating filaggrin expression. In addition, AHR activation induces oxidative stress. However, some AHR ligands simultaneously activate the nuclear factor-erythroid 2-related factor-2 (NRF2) transcription factor, which is a master switch of antioxidative enzymes that neutralizes oxidative stress. The immunoregulatory system governing T-helper 17/22 (Th17/22) and T regulatory cells (Treg) is also regulated by the AHR system. Notably, AHR agonists, such as tapinarof, are currently used as therapeutic agents in psoriasis and atopic dermatitis. In this review, we summarize recent topics on AHR related to atopic dermatitis and psoriasis.

## 1. Introduction

The skin is the outermost surface of the body and is vulnerable to a myriad of external chemicals and internal substances. To maintain homeostasis, skin cells, including keratinocytes, sebocytes, fibroblasts, dendritic cells, and other immune cells, express several chemical sensors, such as aryl hydrocarbon receptor (AHR), pregnane X receptor, constitutive androstane receptor, and peroxisome proliferator-activated receptors [1,2,3,4]. Among these chemical receptors, AHR has gained special attention because it plays a crucial role in photoaging, epidermal differentiation, and immunomodulation [2,3,5,6,7].

AHR, also called dioxin receptor, binds to environmental polyaromatic hydrocarbons and dioxins with high affinity and induces oxidative stress by generating abundant reactive oxygen species (ROS) [5,6,7]. Additionally, AHR is a promiscuous receptor and is activated by a plethora of exogenous and endogenous ligands, such as photo-induced chromophores, phytochemicals, and microbial bioproducts [8,9,10,11,12]. Many AHR ligands exert antioxidative activity by activating antioxidative transcription factor nuclear factor-erythroid 2-related factor-2 (NRF2) [10,13]. Medicinal coal tar and soybean tar Glyteer activate both AHR and NRF2 and have been used to treat inflammatory skin diseases, such as atopic dermatitis (AD) and psoriasis [14,15].

AD and psoriasis are common inflammatory skin diseases. An excellent therapeutic response to biologics indicates a pivotal pathogenic role of interleukin (IL)-4/IL-13 signaling in AD [16,17] and the tumor necrosis factor (TNF)-α/IL-23/IL-17A axis in psoriasis [18,19]. Although distinct signaling pathways operate in developing full-blown AD and psoriasis, 81% of dysregulated genes in AD are shared with those in psoriasis in skin lesions [20]. Notably, recent phase II, randomized dose-finding studies have demonstrated that topical application of the natural AHR agonist tapinarof is efficacious and well tolerated in patients with AD and psoriasis [21,22].

The purpose of this article is to summarize the diverse action of AHR signaling in balancing skin homeostasis and to elucidate the fundamental mechanisms of therapeutic AHR potentials in the treatment of AD and psoriasis.

## 2. AHR Signaling and Modulation of Oxidative and Antioxidative Balance

AHR is a ligand-activated transcription factor [7]. In the absence of ligands, AHR resides in the cytoplasm where it forms a protein complex with heat shock protein 90 (HSP90), hepatitis B virus X-associated protein 2 (XAP-2), and p23 [23,24]. After ligand binding, AHR dissociates from the cytoplasmic complex and a nuclear translocation site of AHR is exposed. Then, AHR is translocated into the nucleus where AHR dimerizes with AHR-nuclear translocator (ARNT), binds DNA responsive elements called xenobiotic responsive elements (XREs), and upregulates the transcription of target genes, such as phase I metabolizing enzyme cytochrome P450 (CYP) members (i.e., *CYP1A1*, *CYP1A2*, and *CYP1B1*) [7,25,26,27,28,29]. 

**Figure 1:**
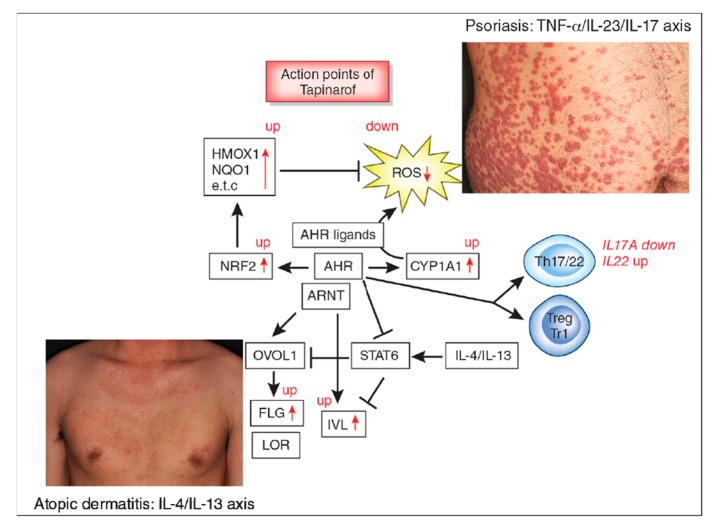
Aryl hydrocarbon receptor (AHR) signal and action points of tapinarof (red words and arrows). AHR is a promiscuous chemical sensor that is activated by various oxidative and antioxidative ligands. Once activated, cytoplasmic AHR translocates into the nucleus where it heterodimerizes with an AHR-nuclear translocator (ARNT) and then induces the transcription of AHR-responsive genes such as cytochrome P450 1A1 (CYP1A1). CYP1A1 degrades AHR ligands. Some ligands such as dioxins are chemically stable and long-lived. Therefore, CYP1A1 generates high amounts of reactive oxygen species (ROS) after sustained efforts to degrade them. Some antioxidative AHR ligands activate nuclear factor-erythroid 2-related factor-2 (NRF2) transcription factor, which upregulates gene expression of various antioxidative enzymes, such as heme oxygenase 1 (HMOX1), NAD(P)H dehydrogenase, and quinone 1 (NQO1), and these antioxidative enzymes neutralize ROS. AHR/ARNT signaling also activates OVO-like 1 (OVOL1) transcription factor and upregulates the expression of filaggrin (FLG) and loricrin (LOR). AHR upregulates the expression of involucrin (IVL) in an OVOL1-independent manner. Therefore, AHR/ARNT signaling accelerates epidermal terminal differentiation and enhances the repair of barrier disruption. Interleukin (IL)-4 and IL-13 activate signal transducer and activator of transcription 6 (STAT6) and inhibit the OVOL1/FLG, OVOL1/LOR, and AHR/IVL axes. However, suitable AHR activation can inhibit the IL-4/IL-13-mediated STAT6 activation and restore the expression of FLG, LOR, and IVL. Regarding immune response, AHR signaling affects T-helper (Th17) differentiation and is essential for IL-22 production. AHR ligation (especially by high concentrations of ligands) induces the differentiation of regulatory cell populations, Treg and Tr1 cells. Tapinarof is an antioxidative AHR ligand and upregulates CYP1A1 expression. Topical tapinarof is efficacious in psoriasis and atopic dermatitis. Current studies demonstrate that tapinarof activates NRF2/antioxidative signaling and reduces oxidative stress. Tapinarof also upregulates FLG and IVL expression. Tapinarof downregulates IL-17A production and increases IL-22 production.

Hazardous dioxins such as 2,3,7,8,-tetrachlorodibenzo-p-dioxin (TCDD) activate AHR and upregulate CYP1A1, CYP1A2, and CYP1B1 expression [5,30,31]. Human keratinocytes abundantly express CYP1A1 and to a lesser extent CYP1B1 but not CYP1A2 [32]. CYP1A1 attempts to metabolize TCDD but the continuous efforts of CYP1A1 are unsuccessful because TCDD is structurally stable [33]. The metabolizing process by CYP1A1 generates excessive amounts of ROS and induces oxidative damage in the cell [5,30,31] (Figure 1). To demonstrate these findings, TCDD-induced ROS production was inhibited in AHR-silenced or CYP1A1-silenced cells [30]. As CYP1B1 silencing did not affect TCDD-induced ROS generation, the AHR-CYP1A1 axis is likely to be crucial for generating cellular oxidative stress by hazardous dioxins [30]. A chemical carcinogen *β*-naphthoflavone also activates CYP1A1 and CYP1A2 via AHR activation in mice [34]. *β*-Naphthoflavone induces mitochondrial ROS generation. However, this activation is attenuated by an AHR inhibitor or CYP1A1/1A2 silencing [34]. AHR-CYP1A1-mediated oxidative stress is responsible at least in part for the production of proinflammatory cytokines, such as interleukin (IL)-1, IL-6, and IL-8 [35,36]. 

To survive during oxidative stress, antioxidative machinery is simultaneously activated after AHR activation in the cells. Ligation of AHR also activates antioxidative transcription factor NRF2 and upregulates the expression of phase II antioxidative enzymes (i.e., glutathione *S*-transferases, heme oxygenase 1 (HMOX1), NAD(P)H dehydrogenase, quinone 1 (NQO1), glutathione S-transferases, and uridine 5’-diphospho-glucuronosyltransferases [13,14,25,26,37,38,39,40]. In contrast to proinflammatory consequences after AHR-CYP1A1-ROS induction, the AHR-NRF2 axis is likely to be anti-inflammatory and reduces the production of proinflammatory cytokines [13,39,41]. Many salubrious antioxidative phytochemical extracts (i.e., artichoke (*Cynara scolymus*) in Mediterranean regions, *Opuntia ficus-indica* in Latin America, and *Houttuynia cordata* in Asia) activate the AHR-NRF2 system and upregulate the expression of antioxidative enzymes [13,37,38]. Dioxins activate the AHR-NRF2 battery [40,42,43], however, their powerful AHR-CYP1A1 activation may induce far more oxidative stress that cannot be extinguished by the AHR-NRF2 antioxidative system. Alternatively, salubrious phytochemical AHR ligands stimulate the AHR-NRF2 axis more strongly than the AHR-CYP1A1-ROS pathway and exert antioxidative action [10].

## 3. AHR and Epidermal Terminal Differentiation

The mammalian epidermis protects the body against injuries from external and environmental factors by providing a barrier-forming cornified layer. Epidermal terminal differentiation or cornified envelope maturation is accomplished by sequential cross-linking of ceramides and various terminal differentiation proteins, such as involucrin (IVL), loricrin (LOR), and filaggrin (FLG) by transglutaminase-1; the majority of these skin barrier-forming proteins map to chromosome 1q21 [44,45].

Notably, activation of the AHR-ARNT axis accelerates epidermal terminal differentiation by coordinately upregulating the production of a series of skin barrier-forming proteins in vivo [46] and in vitro [3,44,47,48]. In parallel, both *Ahr*-deficient and *Ahr*-transgenic mice reveal an abnormality in keratinization [49,50]. Severe abnormalities in keratinization are also observed in *Arnt*-deficient mice [51,52].

Both oxidative and antioxidative ligands for AHR can accelerate epidermal terminal differentiation [3,12,44,47,48]. Slow-metabolizing dioxins induce strong and sustained AHR activation, which results in exaggerated keratinization of keratinocytes and sebocytes and the development of chloracne [2,53]. In contrast, mild and transient AHR activation by antioxidative phytochemical or endogenous AHR ligands are effective in maintaining healthy barrier-intact skin [3,10,54]. 

Sunlight, especially UVB, generates tryptophan photoderivatives such as formylindolo[3,2-b]carbazole (FICZ), which is a high-affinity ligand for AHR that upregulates CYP1A1 expression [8,55,56,57]. Compared with slow-metabolizing TCDD, FICZ is rapidly metabolized by CYP1A1 [8,55,56]. Similar to other AHR ligands, FICZ upregulates filaggrin via AHR signaling [57,58,59]. Although an erythematogenic dose of UVB is harmful through a variety of mechanisms, exposure to a suberythematous dose of UVB prior to tape-stripping results in significantly accelerated barrier recovery rates [60]. Physiological low-dose UVB exposure may be beneficial for skin barrier protection by FICZ-AHR/ARNT-mediated upregulation of filaggrin and other barrier-related proteins [57,58,59] (Figure 1). In this context, topical application of FICZ significantly attenuated transepidermal water loss and dermatitis score in a murine mite-induced dermatitis model [58].

Mechanisms regarding how AHR signaling accelerates keratinocyte differentiation are not fully understood. Kennedy et al. points to an essential role of ROS production in this regulation [47]. We have demonstrated that AHR signaling upregulates the expression of OVO-like 1 (OVOL1) transcription factor and activates its cytoplasmic to nuclear translocation [3,59,61,62]. Both filaggrin and loricrin are under the control of the AHR-OVOL1 pathway, whereas AHR-mediated involucrin upregulation is independent of OVOL1 [63].

**Figure 2:**
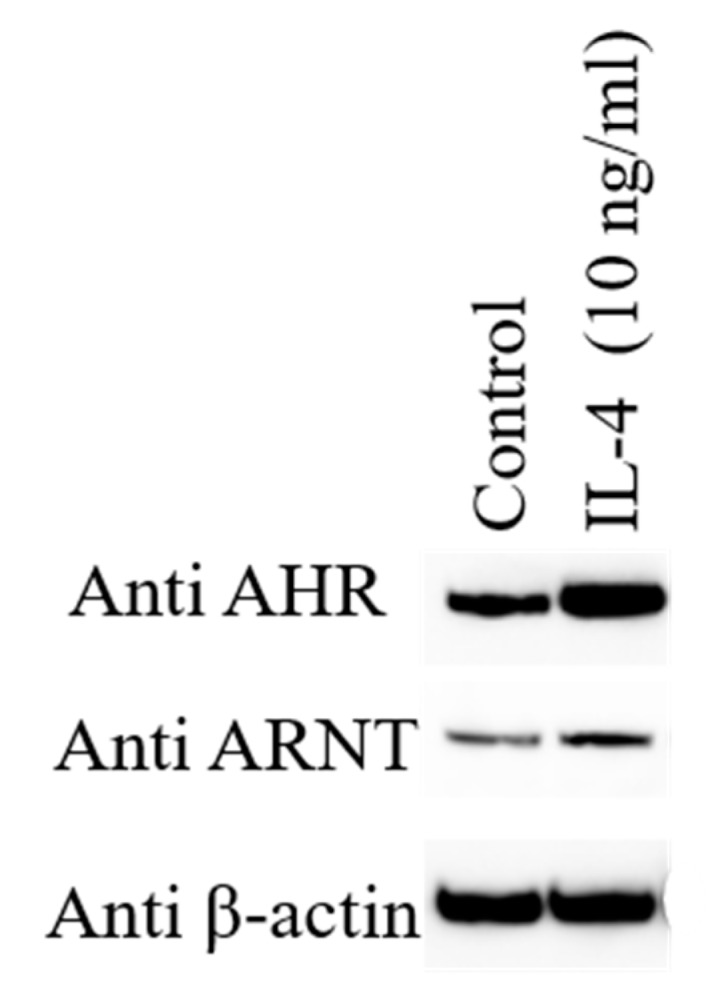
Human epidermal keratinocytes are stimulated with 10 ng/mL of IL-4 augments the protein expression of aryl hydrocarbon receptor (AHR) and AHR-nuclear translocator (ARNT) compared with untreated control by Western blot analysis.

IL-4/IL-13 signaling downregulates the expression of filaggrin, loricrin, and involucrin via signal transducer and activator of transcription 6 (STAT6) activation, impairing the epidermal terminal differentiation and barrier dysfunction [14,15,37,44,61,64,65]. IL-4/IL-13 signaling is likely to impair the cytoplasmic to nuclear translocation of OVOL1, which interferes with the AHR-OVOL1-filaggrin axis [59,61]. Notably, IL-4/IL-13 signaling reciprocally enhances the protein expression of AHR and to a lesser extent ARNT in keratinocytes (Figure 2). Similar results were observed in murine B cells [66]. The implication of IL-4/IL-13-mediated AHR upregulation remains elusive. In addition, IL-4/IL-13-mediated STAT6 activation stimulates keratinocyte to produce periostin, which induces IL-24 production in keratinocytes [67]. IL-24 reduces the filaggrin expression via STAT3 activation [67]. AHR ligands, such as coal tar, Glyteer and FICZ, activate the AHR/ARNT pathway, block the IL-4/IL-13-mediated STAT6 activation, induce the entry of OVOL1 into the nuclei, and restore barrier dysfunction [15,59,61,68].

## 4. AHR and Immune Modulation

As a crucial chemosensor, AHR activity modulates immune function. AHR and its immunological significance are best characterized in intestinal immunology [28,29,69,70]. *Ahr*-deficient mice have an inherent weak gut barrier [71,72,73]. In this context, genome-wide association studies have identified AHR as a susceptibility locus in inflammatory bowel diseases [74]. Indeed, the expression of AHR is reduced in the lesioned intestine in inflammatory bowel diseases [75]. This finding may be strongly related to the fact that the intestinal tract is a rich source of AHR ligands derived from dietary materials and microbial bioproducts [69,76].

Early research on AHR-mediated immune modulation was based on toxicological approaches using dioxins [77]. Dioxin-exposed rodents exhibit waste syndrome, dose-dependent thymic involution, depletion of other lymphoid organs, and reduced circulating lymphocyte counts [77]. Antibody production by B cells is also inhibited by toxic doses of dioxins [77]. However, extensive attention by immunologists has recently been focused on the physiological function of AHR in immune regulation [29,69,70,76].

Ligation of AHR by TCDD and endogenous or natural compounds preferentially affects differentiation and propagation of T-helper 17 (Th17) and T regulatory (Treg) cells [29,69,70]. Tryptophan is an essential amino acid and is thought to produce various candidates for endogenous AHR ligands by different metabolic processes. These metabolic pathways include kynurenine production by indoleamine 2,3-dioxygenase and tryptophan 2,3-dioxygenase, FICZ by UVB exposure, and indole derivatives by bacterial degradation [29,69,70]. Dietary materials such as *Brasicca* contain glucosinolate glucobrassicin, which is metabolized to produce indolo-[3,2-b]-carbazole (ICZ) [29]. The major metabolic pathway of tryptophan is the kynurenine pathway, however, the binding capacity of kynurenine to AHR is very low compared to FICZ and ICZ [29].

In murine CD4^+^ cells, AHR is highly expressed in Th17 cells, not detectable in Th1 and Th2 cells, and marginally expressed in Treg cells [78]. In addition, Lin-Sca^+^ and Sca^−^ progenitor cells in bone marrow, double negative (CD4^−^ and CD8^−^) cells in the thymus, innate lymphoid cell type 3 (ILC3) cells, dendritic cells, γδ T cells, and Langerhans cells express high levels of AHR [28,29]. In *Ahr*-deficient mice, *T-bet* and *Ifng* expression in Th1, *Gata3* and *Il4* expression in Th2, and RORγt (*Rorc*) and *Il17a/Il17f* expression in Th17 cells are not significantly affected. However, *Il22* expression in Th17 cells is almost completely abrogated in *Ahr*-deficient mice [78]. FICZ upregulates *Il17a, Il17f,* and *Il22* expression in Th17 cells. The expression of AHR is detected in human Th17 cells at higher levels than in Th1 cells and FICZ upregulates the *IL17A*, *IL17F,* and *IL22* expression in Th17 cells [78]. Flowcytometric analysis also revealed that FICZ enhances Th17 differentiation and IL-22 production [69]. In a murine Th17-mediated experimental autoimmune encephalomyelitis model, injection of FICZ accelerated disease onset whereas it was delayed in *Ahr*-deficient mice. Treg cells were unaffected in this model [78]. In contrast, TCDD treatment increased the number of Treg cells, which exhibited an immunosuppressive function in a murine graft-versus-host disease model [79]. The TCDD-induced increase in Treg number was abrogated in *Ahr*-deficient mice [79]. These studies suggest that prolonged activation of AHR by TCDD may potentiate Treg cell deviation, but transient AHR activation may shift the immune response toward Th17 and more strongly to Th22 cell differentiation (Figure 1).

Th17 and ILC3 cells express high levels of AHR, IL-17, and IL-22 and are crucial for intestinal protective immunity against commensal and pathogenic microbiota [80]. In contrast to the abovementioned experimental autoimmune encephalomyelitis model in which AHR ligation enhances IL-17 and IL-22 production, *Ahr*-deficiency augments Th17 cell differentiation in the intestinal tract where large amounts of dietary- and microbiota-derived AHR ligands are present [80]. In *Ahr*-deficient mice, the amount of microbiota is significantly increased, which is likely to promote Th17 differentiation. Alternatively, *Ahr*-deficient mice exhibit IL-22 reduction, which is consistently found in an experimental autoimmune encephalomyelitis model. Notably, IL-22 supplementation to *Ahr*-deficient mice normalizes the expansion of the microbiota and reduces Th17 deviation, demonstrating that IL-22 is protective against intestinal infection [80]. In addition, ILC3s produce larger amounts of IL-22 than Th17 cells after AHR ligation [80].

In the gut, heterogenous cell populations exist in Foxp3^+^ Treg cells depending on the expression of neuropilin (Nrp1) and RORγt. Nrp1 is a surface marker to distinguish thymus-derived Tregs (Nrp1 ^+^ tTregs) versus peripherally derived Tregs (Nrp1^-^ pTregs) [70]. In the small and large intestines, all Nrp1 ^+^ RORγt ^−^, Nrp1^−^ RORγt^+^ and Nrp1^−^ RORγt^−^ Treg subpopulations express high levels of AHR [70]. *Ahr* deficiency in these Treg cells induces a significant decrease of Nrp1^−^ RORγt^+^ and Nrp1^-^ RORγt^−^, but not Nrp1^+^ RORγt^−^, Treg subpopulations in the intestine, whereas those in the spleen and mesenteric lymph nodes are not affected [70]. In contrast, AHR activation by FICZ injection preferentially enhances the Nrp1^−^ RORγt^−^ Treg subpopulation. High-throughput RNA sequencing revealed that *Ccr6*, *Gpr15*, *Itgae*, *Rgs9*, and *Gzma* genes important for Treg homing and functions in the gut are downregulated in *Ahr*-deficient Tregs while Th1-associated genes *Ifng*, *Ccl5,* and *Tbx21* are upregulated. Moreover, these AHR-expressing Treg cells inhibit T cell-induced wasting disease and colitis [70].

As described above, AHR ligation induces the CYP1A1 production, which efficiently degrades AHR ligands [28,29]. Therefore, constitutive overexpression of CYP1A1 in mice depletes the reservoir of natural AHR ligands, generating a quasi *Ahr*-deficient state [76]. Th17 cells from *Ahr*-deficient mice do not produce IL-22. In parallel, *Cyp1a1*-overexpressed Th17 cells show a reduced IL-22 production [76]. Both *Ahr*-deficient and *Cyp1a1*-overexpressed mice exhibit loss of ILC3 in the small and large intestines. IL-22 derived from ILC3 and Th17 cells is essential in the defense against *Citrobacter rodentium*. Thus, C. *rodentium*-induced colitis becomes life-threatening both in *Ahr*-deficient and *Cyp1a1*-overexpressed mice [76]. Although AHR ligation upregulates CYP1A1, CYP1B1, and CYP1A2 expression, CYP1B1 and CYP1A2 are not crucial for degrading AHR ligands [76]. FICZ-mediated Th17 differentiation and IL-22 production is achieved by extremely low concentrations of FICZ in *Cyp1a1*-deficient CD4^+^ cells [69]. Furthermore, FICZ promotes IL-17A ^+^ IL-22^+^, but not IL-17 ^+^ IL-22^−^, cell differentiation [69].

Although AHR ligation tends to affect Th17 and Treg cell differentiation, outcomes are inconsistent in different experimental systems. The dose and the duration of AHR activation by high-affinity AHR ligands are likely the primary factors that explain the fate of T cell differentiation [81]. To this end, Ehrlich et al. examined the effects of low and high doses of four high-affinity AHR ligands (TCDD, FICZ, 2-(1H-Indol-3-ylcarbonyl)-4-thiazolecarboxylic acid (ITE), and 11-Chloro-7H-benzo[de]benzo[4,5]imidazo[2,1-a]isoquinolin-7-one (11-Cl-BBQ)) on CD4^+^ T cell differentiation using a parent-into-F1 alloresponse mouse model. Intraperitoneal injection of high doses of all agents induced the production of IL-10 producing, Foxp3^+^ type 1 regulatory T cells (Tr1 cells) on day 2, and increased Foxp3^+^ Tregs on day 10 in conjunction with suppression of the alloresponse. Alternatively, low doses of the ligands, even when given daily, did not induce Tregs nor alter the alloresponse, but instead increased the percentage of CD4^+^ cells that produce IL-17 [81]. In summary, accumulating evidence suggests that AHR ligation stimulates Th17 cells to differentiate into Th17/22 cells. AHR ligands may also enhance the regulatory cell population especially in high doses.

## 5. AHR and Atopic Dermatitis

AD is a common and heterogenous eczematous skin disorder characterized by Th2-deviated skin inflammation, barrier disruption, and chronic pruritus [17,82,83]. Frequent relapse with intense pruritus deteriorates quality of life and decreases treatment satisfaction of the afflicted patients [84,85,86,87,88]. The lifetime incidence of AD is as high as 20% in the general population [89]. Skin barrier dysfunction is associated with the reduced production of terminal differentiation molecules such as filaggrin [15,51]. Abnormal skin barrier integrity also causes an increased colonization of microbes such as *Staphylococcus aureus,* which further exacerbate Th2-deviated skin inflammation [90,91]. In addition, some autoimmune diseases are comorbid with AD [92].

Investigation on *AHR* gene polymorphism reveals that *AHR* rs10249788 and rs2066853 polymorphisms are found in patients with AD, psoriasis, and healthy controls, but no significant differences were detected in genotype or allele frequencies between the three groups [93]. However, the *AHR* rs2066853 (AG + AA) or rs10249788 (CT + TT) genotypes are a risk factor for severe dry skin phenotype and the combined rs10249788 (CT + TT) and rs2066853 (AG + AA) genotypes lead to a higher risk for severe dry skin in Chinese patients with AD [93]. rs10249788 exists in the AHR promoter region where nuclear factor 1C (NF1C) binds and suppresses the transcription and protein expression of AHR [94]. Notably, NF1C prefers to associate with the C allele compared to the T allele at rs10249788. Thus, subjects with the rs10249788 (CC) allele express less AHR than those with the rs10249788 (TT) allele [94]. In fact, AHR mRNA levels for the TT genotype are 1.7-fold higher than those for the CC genotype [95]. No significant differences were obtained in AHR production between the CC and CT genotypes [95]. In parallel with increased levels of AHR, cells with the TT genotype express significantly higher levels of CYP1A1, IL-24, and IL-1β [95]. It is intriguing that IL-24 downregulates the filaggrin expression via STAT3 activation [67].

Immunohistological and real time PCR studies for AHR have been reported in AD [96,97]. Hong et al. showed an increased expression of both AHR and ARNT without CYP1A1 induction in the lesioned skin of AD compared with normal control skin [96]. Alternatively, Kim et al. demonstrated an increased expression of ARNT and CYP1A1 but not AHR in the lesional skin of AD [97]. As the Th2-deviated milieu potently reduces filaggrin and other barrier-related molecules, the upregulation of AHR/ARNT may be compensatory to attenuate the Th2-mediated filaggrin reduction. A recent study by Yu et al. demonstrated the possibility that the Th2-deviated milieu decreases the production of endogenous AHR ligand such as indole-3-aldehyde by commensal skin microbiota [98]. These findings collectively suggest that most AHR likely lack physiological ligands in the Th2-prone milieu in AD. Therefore, rapid-metabolizing AHR ligands, such as FICZ and indole-3-aldehyde, appropriately activate the AHR/ARNT/FLG axis and may be beneficial in treating AD [58,98]. However, vigorous and long-lasting activation of the AHR/ARNT/FLG axis by slow-metabolizing dioxins and environmental pollutants may exacerbate barrier dysfunction and aggravate AD [96,99].

Although the pathogenic implication of AHR and its gene polymorphism in AD remain elusive, recent clinical trials using topical AHR ligand tapinarof have reported its efficacy for AD [100,101,102]. Tapinarof (5-[(E)-2-phenylethenyl]-2-[propan-2-yl] benzene-1, 3-diol, WBI-1001, GSK2894512 or bentivimod) is a naturally derived (but is now a fully synthetic) hydroxylated stilbene produced by bacterial symbionts of entomopathogenic nematodes [100,101,102,103]. Tapinarof is a high affinity AHR ligand with antioxidative activity via NRF2 activation and a ROS-scavenging structure [102] (Figure 1). Tapinarof has gained increased attention because its topical application is efficacious for patients with AD in clinical trials [21,100,104]. Tapinarof activates the AHR/CYP1A1 axis and augments the expression of filaggrin and involucrin [102]. Even in barrier-disrupted AD patients, systemic absorption of topical tapinarof is limited and likely decreases during the treatment course in parallel with treatment success that restores the barrier dysfunction [104]. In general, topical tapinarof is tolerable but frequent adverse events include headaches and folliculitis [104].

In an early clinical trial, patients with AD affecting 3–20% of their body surface area (BSA) and with an Investigator’s Global Assessment (IGA; 0: clear, 1: almost clear, 2: mild, 3: moderate, 4: severe, 5: very severe) of 2–4 were randomized (1:1:1) to receive a placebo (*n* = 51), topical tapinarof 0.5% (*n* = 50) or 1% (*n* = 47) in a cream formulation applied twice daily for six weeks [100]. There was a decrease of 1.3 (43%; *p* < 0.001; 95% confidence interval (CI) −1.2 to −0.5) and 1.8 (56.3%; *p* < 0.001; 95% CI −1.6 to −0.9) in IGA at day 42 in the topical tapinarof 0.5% and 1% groups, respectively, compared with a decrease of 0.5 (14.7%) in the placebo group. At day 42, improvement in Eczema Area and Severity Index (EASI) score was 68.9% (*p* < 0.001) and 76.3% (*p* < 0.001) for tapinarof 0.5% and 1%, respectively, compared with 23.3% for placebo. Improvement in pruritus severity score at day 42 was 29.8% (*p* < 0.001) and 66.9% (*p* < 0.001) for tapinarof 0.5% and 1%, respectively, compared with 9.5% for placebo [100]. Adverse events included headaches (placebo: 0%; 0.5% tapinarof: 8%; 1% tapinarof: 14%), migraines (placebo: 0%; 0.5% tapinarof: 4%; 1% tapinarof: 3%), folliculitis (placebo: 0%; 0.5% tapinarof: 6%; 1% tapinarof: 8%), and contact dermatitis (placebo: 0%; 0.5% tapinarof: 3%: 1% tapinarof: 5%) [100].

A phase II, double-blind, vehicle-controlled, randomized, six-arm trial (1:1:1:1:1:1) in patients aged 12 to 65 years, with BSA involvement of at least 5% to 35% and an IGA score of 3 or higher (moderate to severe) at baseline was performed. Primary end points included an IGA score of clear or almost clear (0 or 1) and a minimum two-grade improvement (treatment success) at week 12 [21]. The rates of treatment success with topical tapinarof cream at week 12 were 53% (1% twice daily, *n* = 40), 46% (1% once daily, *n* = 41), 37% (0.5% twice daily, *n* = 43), 34% (0.5% once daily, *n* = 41), 24% (vehicle twice daily, *n* = 42), and 28% (vehicle once daily, *n* = 40). The rate with tapinarof 1% twice daily (53%) was statistically significantly higher than the rate with vehicle twice daily (24%). Notably, treatment success was maintained for four weeks after the end of tapinarof treatment. The proportion of patients achieving EASI75 (75% or greater improvement in EASI) score reduction at week 12 was significantly higher in the groups treated with 1% tapinarof (60% and 51% for twice daily and once daily, respectively) than with vehicle (26% and 25% in the groups receiving vehicle twice daily and once daily, respectively) [21]. Headaches (e.g., 10% (1% twice daily), 2% (0.5% twice daily), and 0% (0.5% twice daily)) and folliculitis (e.g., 10% (1% twice daily), 7% (0.5% twice daily), and 0% (0.5% twice daily)) were again frequent adverse events [21].

In a murine dermatitis model, topically applied FICZ activated AHR and significantly reduced the dermatitis score and histological inflammation with a decrease of *Il22* gene expression in chronic mite antigen-induced dermatitis [58]. In addition, topical FICZ restored the dermatitis-induced filaggrin downregulation [58]. CCL17 and CCL22 are crucial chemokines to recruit Th2 cells [68]. IL-4/IL-13 stimulates dendritic cells to produce CCL17 and CCL22 via STAT6 activation and contributes to the recruitment of Th2 cells in the lesional skin of AD [68]. Soybean tar Glyteer inhibits the IL-4/IL-13-mediated STAT6 activation and subsequent production of CCL17 and CCL22 in dendritic cells [68]. In addition, pruritogenic Th2 cytokine IL-31 synergistically upregulates the IL-4/IL-13-mediated CCL17 and CCL22 production in dendritic cells because IL-4/IL-13 increase IL-31 receptor A (IL31RA) expression [105]. Glyteer again attenuates the IL-4/IL-13-mediated IL31RA upregulation and subsequent CCL17 and CCL22 production by inhibiting STAT6 activation [105]. It is known that coal tar inhibits STAT6 activation via the NRF2-antioxidative pathway [15]. Ligation of AHR by FICZ also reduces the expression of type 1 IgE Fc receptor in Langerhans cells [106].

Although antioxidative AHR ligands are therapeutic for dermatitis, exaggerated activation of AHR by genetic manipulation in transgenic mice or by dioxin treatment induces itchy dermatitis most likely due to an abnormally accelerated keratinization process, epidermal acanthosis, elongation of nerve fibers, and production of pruritogenic artemin [47,99,107]. Therefore, extreme activation of AHR is deleterious for skin. In parallel, ovalbumin-induced delayed hypersensitivity is enhanced by topical benzopyrene with upregulation of IL-5, IL-13, and IL-17 expression in lymph node cells [96].

Since FICZ is an endogenous UVB photoproduct [8], the barrier-protecting effects of FICZ may explain, at least in part, why UVB phototherapy is efficacious for the treatment of AD and psoriasis [108,109].

## 6. AHR and Psoriasis

Psoriasis is an (auto)immune-mediated disease that manifests as widespread desquamative erythema [110,111]. Males are twice as likely to be affected than females [112,113]. The cosmetic disfigurement associated with psoriasis profoundly impairs the patients’ quality of life, treatment satisfaction and adherence, and socioeconomic stability [114,115]. The autoimmune nature of psoriasis is exemplified by its high comorbidity with psoriatic arthritis [110,116,117,118] and other autoimmune diseases including autoimmune bullous diseases [119,120,121,122,123,124]. Psoriasis is also comorbid with cardiovascular diseases, metabolic diseases, and renal disorders, which represent a condition called inflammatory skin march [111,125,126,127,128,129]. The excellent therapeutic efficacy of anti-TNF-α/IL-23/IL-17A biologics for psoriasis point to the central role of the TNF-α/IL-23/IL-17A axis in its pathogenesis [18,19,130,131,132,133,134] Additionally, genetic and environmental factors are known to be involved in its pathogenesis [135,136].

As AHR predominantly regulates the immune balance of Th17/22 and Treg cells [28,29,69,70], AHR is expected to play a significant role in psoriasis [102]. In an imiquimod-induced psoriasis model, *AhR* deficiency exacerbates skin inflammation with upregulated gene expression of *Il22*, *Il17a,* and *Il23* [137]. The intensity of delayed type-hypersensitivity is also enhanced in *Ahr*-deficient mice [137]. However, further experiments demonstrated that *Ahr*-deficiency in nonhematopoietic cells, including keratinocytes, but not in hematopoietic cells, was likely responsible for the exacerbation of inflammation [137]. Notably, intraperitoneal injection of FICZ ameliorated the imiquimod-induced psoriasis-like inflammation. Tapinarof and FICZ also reduced the imiquimod-induced psoriasiform skin inflammation by inhibiting *Il17a*, *Il17f*, *Il19*, *Il22*, *Il23a*, and *Il1b* gene expression [102]. The therapeutic action of tapinarof and FICZ was AHR-dependent because it was not observed in *Ahr*-deficient mice [102]. In an ex vivo activation assay of skin-resident immunocompetent cells using normal human skin, tapinarof inhibited the expression of *IL17A* message approximately 50% but increased the *IL22* expression [102,138] (Figure 1). In mice, IL-22 is produced from Th17, γδT, ILC3, and CD4^−^CD8^−^TCRβ^+^ cells [139]. AHR was required for IL-22 production by Th17, but not by the three other cell types, in the imiquimod-treated ears [139]. Although imiquimod-induced skin inflammation is popular as a psoriasis model, attention should be paid because imiquimod is degraded by CYP1A1 so the efficacy of AHR agonists may partly rely on this effect in the imiquimod model [140].

Immunohistological and real time PCR studies have demonstrated that the expression of AHR and ARNT is upregulated in the lesional skin of psoriasis, whereas CYP1A1 expression was significantly decreased compared to normal controls [97]. In contrast, serum levels of both AHR and CYP1A1 are elevated in patients with psoriasis compared to normal controls [141]. Further studies are warranted to investigate these controversial data.

In parallel with its preclinical studies, topical tapinarof is efficacious in the treatment of psoriasis. In a randomized, double-blind, placebo-controlled phase II trial, 61 patients with 1–10% BSA covered with plaque psoriasis and a PGA of 2–4 were randomized (2:1) to receive either 1% tapinarof cream or placebo, applied twice daily for 12 weeks [142]. At week 12, the improvement in PGA was 62.8% for patients treated with tapinarof compared with 13.0% for patients randomized to placebo (*p* < 0.0001). The proportion of patients who achieved a PGA of clear or almost clear was significantly higher with tapinarof treatment (67.5%) compared with placebo (4.8%, *p* < 0.0001) [142]. In another double-blind, vehicle-controlled, randomized six-arm trial (1:1:1:1:1:1) in adults with psoriasis with body surface involvement ≥ 1% and ≤ 15% and PGA score ≥ 2 at baseline, treatment success defined by PGA 0 or 1 and a two-grade improvement at week 12 was significantly higher in the tapinarof groups (65% (1% twice daily), 56% (1% once daily), 46% (0.5% twice daily), and 36% (0.5% once daily)) than vehicle groups (11% (twice daily) and 5% (once daily)); this was maintained for four weeks post-treatment [22]. The most commonly (≥5%) reported adverse events that emerged after treatment were folliculitis (19/152, 13% tapinarof groups and 1/75, 1% vehicle groups) and contact dermatitis (12/152, 8% only in the tapinarof groups) [22]. These preclinical and clinical studies reinforce that AHR ligand tapinarof is efficacious in the treatment of psoriasis and atopic dermatitis. In 2019, tapinarof (1% benvitimod cream) was officially approved by the Chinese government for medical use for psoriasis after successful Chinese clinical trials [103]. Overall, why topical and systemic AHR ligands reduce psoriatic inflammation by inhibiting IL-17 and IL-22 in vivo while the same ligands upregulate the expression of IL-17 and IL-22 in vitro is still unknown.

## 7. Conclusions

Humans empirically utilize natural antioxidative resources to keep their skin healthy, including coal tar, *Galactomyces* fermentation filtrate, *Opuntia ficus-indica* in Latin America, Artichoke in Mediterranean regions, and *Houttuynia cordata* and *Artemisia princeps* in Asia [12,13,15,37,38,62]. These agents are potent AHR ligands which activate the AHR-ARNT system and enhance the terminal differentiation of epidermal keratinocytes [12,13,15,37,38,62]. They also exert antioxidative action via AHR-NRF2 activation [12,13,15,37,38]. The study of the signal transduction mechanisms of the AHR/ARNT system has demonstrated that this system is also deeply involved in immune regulation especially in Th17/22 and Treg maturation [28,29]. A selective AHR agonist, tapinarof is currently being studied because this medicinal agent improves both psoriasis and AD in which different pathomechanisms operate (the TNF-α/IL-23/IL-17 axis in psoriasis and IL-4/IL-13 signaling in AD). Further mechanistic approaches are warranted to develop new drugs targeting the AHR system.
